# Commentary: Molecular Mechanisms of Action of FSH

**DOI:** 10.3389/fendo.2019.00593

**Published:** 2019-08-27

**Authors:** Rossella Cannarella, Rosita A. Condorelli, Sandro La Vignera, Aldo E. Calogero

**Affiliations:** Department of Clinical and Experimental Medicine, University of Catania, Catania, Italy

**Keywords:** FSH, IGF1, IGF-1 R, gonadal function, signaling

We have carefully read and appreciated the recent article by Casarini and Crépieux, entitled “Molecular mechanisms of action of FSH” (1), which provides a clear and exhaustive overview of the FSH signaling pathway. The authors notably proposed a comprehensive outline of the molecular mechanisms of FSH signaling, including the cross-talk between FSH-dependent steroidogenic, life, and death signals in granulosa cells and the temporal succession across the cAMP/PKA pathway. The authors concluded that the full comprehension of the FSH-mediated signaling action is of high physiological and clinical relevance and admit that the FSH signaling pathway is not entirely deciphered.

We agree with this statement and we would like to point out that other molecules are involved in the complex role that FSH plays in the gonadal function. Particularly, insulin-like growth factor 1 (IGF1) and its receptor (IGF1R) have already been showed to act in FSH signaling in granulosa cells (2, 3). Similarly, we recently confirmed such evidence in porcine Sertoli cells (4).

We have focused on this topic starting from the presence of oligozoospermia in a patient with chromosome 15q26.3 duplication involving the *IGF1R* gene (5). *In-vitro* and *in-vivo* data from both non-mammalian and mammalian species support the role of IGF1 in Sertoli cell proliferation and in germ cell proliferation and differentiation, as well as in testis differentiation (6). The expression of the *Igf1r* has been shown to be required for FSH responsiveness (7) in granulosa cells, thus pointing out to a possible role for the IGF1R in FSH signaling pathway. The specific molecular mechanism through which a coupled G protein receptor (CGPR) such as the FSHR involves a tyrosine kinase receptor such as the IGF1R in its signaling pathway has been explored in granulosa cells (8). The authors have shown that the two receptors are linked by the same molecular pathway and the insulin receptor substrate 1 (IRS1) is the hub-linking (8).

We have recently shown the involvement of IGF1R in the FSH signaling pathway in porcine Sertoli cells. In these primary cultures, inhibition of the IGF1R affects the FSH capacity to phosphorylate target proteins (4). Similarly to FSH, IGF1 down-regulates *antiMullerian hormone (AMH)* gene expression and protein secretion, and enhances those of inhibin B (9). Co-incubation with both IGF1 and FSH results in a higher suppression of AMH secretion compared to that obtained with FSH alone (9). This likely suggests an enhancing effect of IGF1 on FSH. Furthermore, differently from FSH, IGF1 stimulates Sertoli cell proliferation, whereas FSH was able to stimulate cell proliferation only when co-administered with IGF1 (9).

IRS1 is linked with the intracellular domain of the IGF1R and plays a role in the FSH pathway (5, 8). Fascinatingly, it also interacts with the intracellular domain of the insulin receptor (IR) and is involved in the pathogenesis of insulin-resistance when abnormally phosphorylated (10). This may point to hypothesize a possible interference in FSH responsiveness in patients with insulin-resistance. We found that exposure to insulin affects the FSH-induced inhibin B secretion from porcine Sertoli cells, lowering the peak of secretion, consistent with a possible interference of insulin with the FSH action (11).

In conclusion, data from both granulosa and Sertoli cells support the role of IGF1R is the FSH signaling pathway. This acknowledgment is of great importance since it might help in the elucidation of apparently idiopathic form of male infertility or FSH unresponsiveness.

## Author Contributions

All authors listed have made a substantial, direct and intellectual contribution to the work, and approved it for publication.

### Conflict of Interest Statement

The authors declare that the research was conducted in the absence of any commercial or financial relationships that could be construed as a potential conflict of interest.
